# P-692. Trends in Pediatric Respiratory Virus Detection Using a Rapid, Accessible Multiplex real-time PCR assay in the Outpatient Setting: May 2024 – March 2025

**DOI:** 10.1093/ofid/ofaf695.905

**Published:** 2026-01-11

**Authors:** Licita Schreiber, Michal Tenenbaum, Tamar Shapira, Shirley Shapiro Ben David

**Affiliations:** Maccabi Healthcare Services, Mega Lab, Rehovot, HaMerkaz, Israel; Maccabi Healthcare Services, Mega Lab, Rehovot, HaMerkaz, Israel; Maccabi Healthcare Services, Mega Lab, Rehovot, HaMerkaz, Israel; Maccabi Healthcare Services, Tel Aviv, Tel Aviv, Israel

## Abstract

**Background:**

Accurate and timely detection of respiratory viruses is essential for effective patient management and informed public health decision-making. In Israel, such diagnostics are typically hospital-based. This study describes the first 11 months of a rapid, multiplex real-time PCR assay introduced in community clinics, offering high accuracy and same-day results within a large health maintenance organization (HMO).

**Figure 1:**
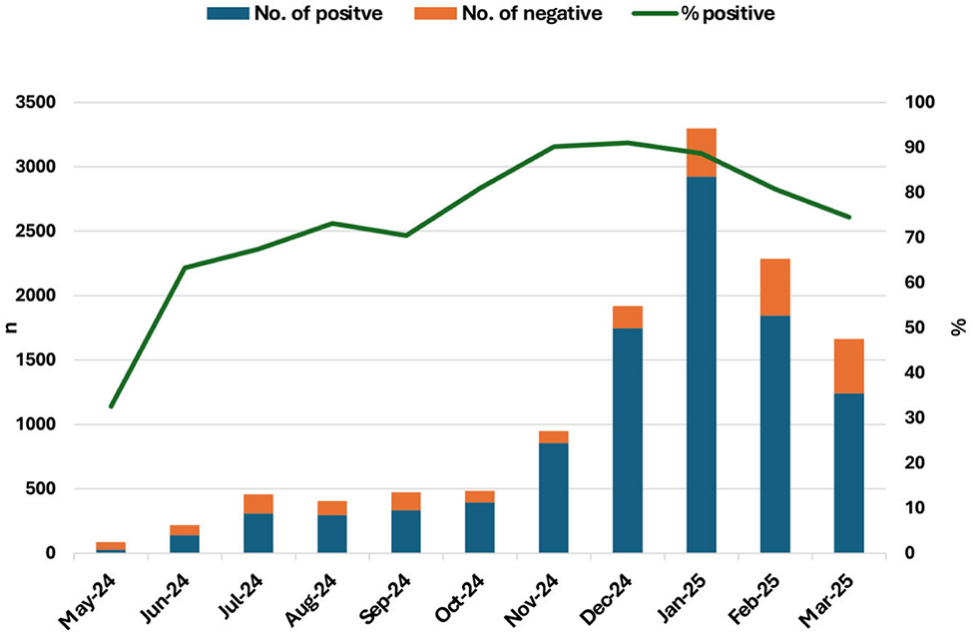
Trends in Positive and Negative Viral Respiratory PCR Tests During the study period, among children aged 0-18 years, May 2024- March 2025

**Figure:**
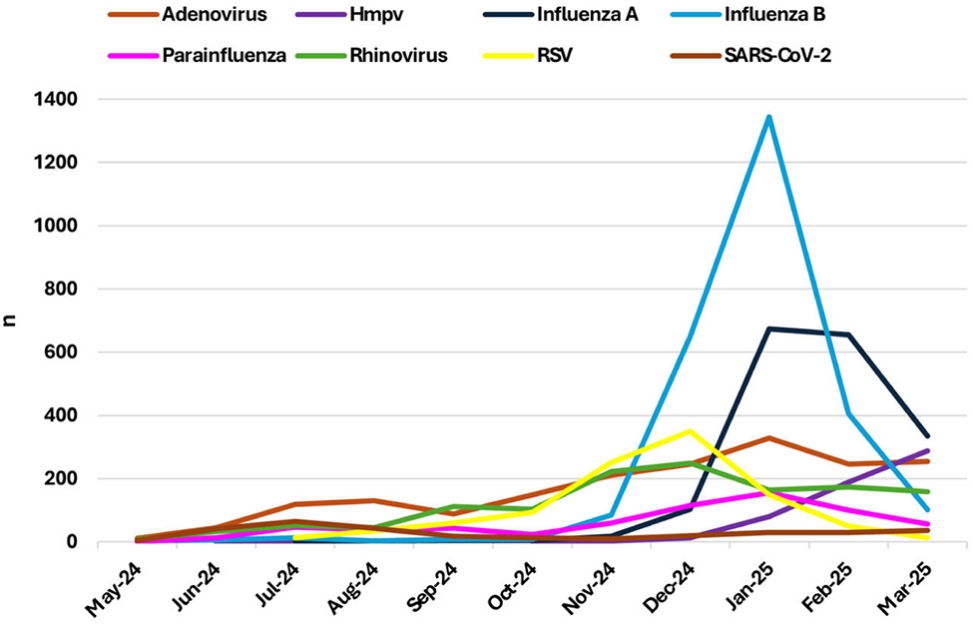
Trends of respiratory virus detection among children aged 0-18 years, May 2024- March 2025

**Methods:**

This observational study was conducted within Maccabi Healthcare Services, a large outpatient HMO in Israel that provides care to over 2.7 million patients—about a quarter of the country's population, nationwide. Between May 2024 and March 2025, children under the age of 18 presenting to community clinics were tested for respiratory pathogens at the discretion of the treating physician. TheAllplex RV Master Assay simultaneously detects eight viruses: SARS-CoV-2, Human parainfluenza virus, Influenza A and B virus, Adenovirus, Rhinovirus, Human metapneumovirus (HMPV), RSV and included internal control. Monthly positivity rates were calculated for each virus.

**Figure:**
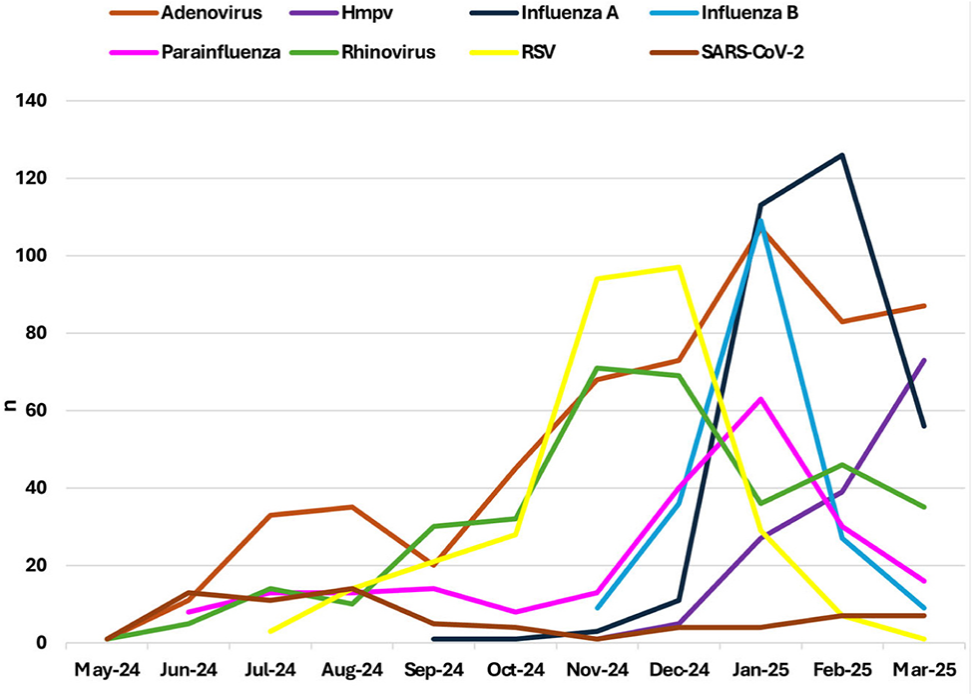
Trends of respiratory virus detection among infant aged 0-12 months, May 2024- March 2025

**Results:**

A total of 12,243 respiratory panel tests were performed, with monthly volume rising from 86 in May 2024 to 3,298 in January 2025 (Figure 1). Of them, 10,109 tests were positive, including 2,144 among infants below one year of age. Influenza B peaked in January (1,345 cases), while influenza A peaked in January–February with lower counts (674 and 655, respectively; Figure 2a). RSV activity was highest in November–December (350 and 251, respectively), especially among infants under 12 months (94 and 97, respectively; Figure 2b). HMPV rose steadily, peaking in March with 288 cases, including 73 infants. SARS-CoV-2 peaked in July (65 cases). Adenovirus was consistently detected throughout, peaking at 328 cases in January, including 107 infants.

**Conclusion:**

This study describes pediatric respiratory virus trends following the outpatient introduction of a rapid multiplex PCR assay. Influenza B peaked sharply in winter, while influenza A showed moderate activity later. RSV was prominent in infants under one year during late fall. Further research is needed to evaluate its impact on viral burden and antibiotic use.

**Disclosures:**

Shirley Shapiro Ben David, MD, Astra zenica: Board Member|Astra zenica: Honoraria|Giliad: Honoraria|GSK: Board Member|GSK: Honoraria|MSD: Board Member|MSD: Honoraria|Pfizer: Grant/Research Support|Pfizer: Honoraria

